# Trauma training course: innovative teaching models and methods for training health workers in active conflict zones of Eastern Myanmar

**DOI:** 10.1186/s12245-014-0046-z

**Published:** 2014-12-12

**Authors:** Charles H Washington, Francis J Tyler, Julia Davis, Douglas R Shapiro, Adam Richards, Matthew Richard, Thomas J Lee, Taryn L Colton, Louis Berk, Loren Rauch, Eh Kalu Shwe Oo, Richard Hahn, Lawrence M Stock

**Affiliations:** Department of Epidemiology, Bloomberg School of Public Health, Johns Hopkins University, 615 N. Wolfe Street, Baltimore, MD 21205 USA; Access Aid International, PO box 6086, St Kilda Road, Melbourne, VIC 3004 Australia; Community Partners International, 2550 Ninth Street, Suite 111, Berkeley, CA 94710 USA; Ross University School of Medicine, PO box 266, Roseau, West Indies Commonwealth of Dominica; Division of General Internal Medicine & Health Services Research at UCLA, 911 Broxton Plaza, Los Angeles, CA 90024 USA; David Geffen School of Medicine at UCLA, 10833 Le Conte Avenue, Los Angeles, CA 90095 USA; University of Arizona College of Medicine, 1501 N. Campbell Avenue, Tucson, AZ 85724 USA; University of Massachusetts Medical School, 55 Lake Avenue North, Worcester, MA 01655 USA; Antelope Valley Hospital, 1600 W Avenue J, Lancaster, CA 93534 USA; Karen Department of Health and Welfare, Mae Sot, Tak Province Thailand; High Desert Health Systems, Los Angeles County Department of Health Services, 44900 60th Street West, Lancaster, CA 93536 USA

**Keywords:** Trauma training, Simulation, Health worker, Myanmar, Conflict

## Abstract

**Background:**

Myanmar has struggled through decades of internal conflict, which has negatively impacted the country’s health outcomes. Recent government changes have brought hope and reduced conflict. The ethnic minority groups have suffered the brunt of the health consequences and reside in regions that lack health infrastructure, resources, and providers. Due to the chronic lack of healthcare providers within conflict areas, health workers (HWs) have been trained in an effort to fill the void. Research has shown that these non-physician clinicians positively impact health outcomes in developing countries. These HWs are supported by community-based organizations in collaboration with foreign non-governmental organizations. Started in 2000, the trauma training course was developed to meet the educational needs of these HWs.

**Methods:**

Essential procedures for HWs in conflict zones were identified, and teaching methods were adapted to develop models that were simple, reproducible, cost effective, and able to facilitate effective learning within the limitations of these challenging environments. This paper presents simulation models developed to teach trauma injury evaluation and management in resource-limited settings to HWs.

**Results:**

Material and construction of the models described include breathing, chest, cricothyroidotomy, circulation, wound repair, fracture/dislocation, splinting, fasciotomy/amputation, and an animal model. In 2013, a pre/post test and post-training evaluation were completed, which demonstrated an increase in understanding of the material and satisfaction with the training.

**Conclusions:**

The simulation models described engage the HWs in clinical skills practice specific to injury management, which builds upon the HWs existing knowledge and facilitates an increased understanding of life-saving procedures. Through observation of the HW performance and HW feedback, these simulation models have increased the understanding of trauma management. Limitations include lack of a graduated learning system for the HWs, logistics, and time constraints. Despite the barriers faced, we feel that this is a necessary program that has reduced morbidity and mortality due to traumatic injury in the geographic areas that the HWs serve. With the changing political environment in Myanmar and the development of peace agreements between the government and the ethnic minority groups, these HWs can be integrated into Myanmar’s evolving health system.

## Background

Myanmar (formerly known as Burma) has struggled through more than 60 years of internal conflict, which has negatively impacted the country’s health outcomes. In 2000, the World Health Organization ranked Myanmar’s health system 190th out of 191 countries [1]. In addition, decades of human rights abuses have largely contributed to the country’s consistently poor health outcomes [2-4]. Recent government changes, including a democratically elected president and peace agreements between the Myanmar military and ethnic minority groups, have brought hope and reduced armed conflict [5-7].

The ethnic minority groups have suffered the brunt of the conflict, abuses, and health consequences [4,8]. While dispersed throughout the country, the largest ethnic minority populations reside in Myanmar’s border regions. In these regions there is a lack of health infrastructure, resources, providers, and an abundance of internally displaced persons (IDPs; an estimated 429,000 people in 2013) [2,3,9,10]. This has lead to a chronic state of health crisis in these areas. For example, in 2003, estimates of under-5 mortality rates were 26, 107, and 276 per 1,000 live births, respectively, for Thailand, Myanmar, and eastern Myanmar (a predominately ethnic minority area) [8,11]. Landmine and gunshot wounds compose a majority of the trauma-related injuries [12].

Due to the chronic lack of healthcare providers within conflict areas of Myanmar, health workers (HWs) have been trained in an effort to fill the void [3,8,12]. Research has shown that these non-physician clinicians, if appropriately educated and trained, positively impact health outcomes in developing countries [12-14]. These HWs are largely supported and receive training from local community-based organizations (CBOs) such as the Mae Tao Clinic, Karen Department of Health and Welfare (KDHW), the Backpack Health Worker Teams (BPHWT), and the Shan Health Committee who have acted as a crucial part of Myanmar’s non-government associated health care system with fixed and mobile clinics [3,12]. Additional trainings occur at camps with IDPs and refugees in Thailand. The duration of initial training ranges from 4 to 18 months and includes primary care, infectious disease, maternal-child care, first aid, trauma, and public health [12]. These CBOs in cooperation with foreign non-governmental organizations (NGOs) have been working collaboratively over the years to increase capacity through trainings, project development, drug procurement, infrastructure building, and fundraising to support this healthcare system [12].

A USA-based NGO began working in eastern Myanmar in 1998 with a focus on public health initiatives and training in areas such as malaria, women’s health, lymphatic filariasis, and trauma. In 2000, in partnership with the KDHW, the trauma training course (TTC) began with a focus on basic trauma knowledge and skills to address trauma morbidity and mortality in eastern Myanmar [12]. KDHW has since provided program management, including supplies, medication, equipment, data collection and analysis, and fundraising. The TTC has evolved to meet the demands of the resource-limited settings and educational needs of the HWs. The TTC has developed from a lecture-dominated format to one focused on simulation. Over the past 9 years, the TTC has trained approximately 395 community health workers. Table 1 lists the topics covered during the TTC.

**Table 1:** **Topics covered by the TTC**

**Topics**	
Amputation	Primary survey
Anatomy and physiology	Recognition and management of airway and breathing injuries
Antibiotic use	Recognition and management of shock
Bleeding management	Rehabilitation
Health worker safety/situational awareness	Resuscitation
Limb injury management including orthopedic care	Safe field blood transfusions
Local anesthetics	Secondary trauma survey
Mass casualty	Sedation
Mortality/morbidity reducing procedures	Suturing
Nutrition	Trauma in special populations (children, pregnant, elderly)
Compartment syndrome identification and management with fasciotomy	Trauma mental health care
	Wound care

There has been an increased emphasis on simulation throughout the development of the TTC. Simulation is used throughout medical education [15-19]. Benefits of simulation training include the opportunity to learn and practice in a safe environment without risks to the student or patient and the ability for instructors to provide feedback in a reduced stress and danger-free environment [15,16,19-21]. Studies have shown improved surgical skills through simulation in a variety of settings [22,23]. In rural areas, training using simulation models is of particular interest because traditional clinical training methods are often not available.

While it is widely accepted that simulation-based education and training has many advantages, creating a medical model that closely resembles its clinical counterpart can be challenging. In resource-limited settings, competing financial priorities necessitate low-cost medical models. Our trainers have worked over the years to develop and refine medical models that are simple, reproducible, cost effective, and able to facilitate effective learning within the limitations of these challenging environments. This paper presents simulation models developed to teach trauma injury evaluation and management in resource-limited settings to HWs.

## Methods

Essential procedures for HWs in a resource-limited and conflict setting were identified. Over the last 13 years, clinical simulation models have been borrowed, developed, and refined to make certain the models depict the basic concepts necessary for diagnosis and management of traumatic injury. Literature from Maurice King (Primary Surgery, Oxford University Press, New York, USA), Hans Husum, (Save Lives, Save Limbs, Third World Pres, Ritoe, Norway), and the American College of Surgeons (Advanced Trauma Life Support, Illinois, USA), in addition to the decades of experience of our staff, have served as cornerstones for the simulation models and training concepts. From the start, there was a focus on building low-cost models with locally available resources or items easily brought from abroad.

The TTC is organized into training modules, each focusing on a particular essential procedure. All training modules begin with a review of anatomy and physiology, followed by discussion on injury patterns, diagnosis, and management. Simulation models are used to engage the HWs and to further facilitate an understanding of the anatomy, physiology, and management of injuries. Each training module is supported by a single or multiple simulation models, ultimately leading to an animal model that incorporates the skills and concepts from the non-animal models utilized throughout the course.

To gain insight into the HWs conceptualization of the skills learned on simulation models to live patients, HWs were asked to provide a summary of their cases in the field from the prior year. Additionally, a post-training evaluation and pre/post test were completed by the 26 HWs that attended the TTC in 2013.

## Results and discussion

### Training models

#### Breathing model

**Figure 1:**
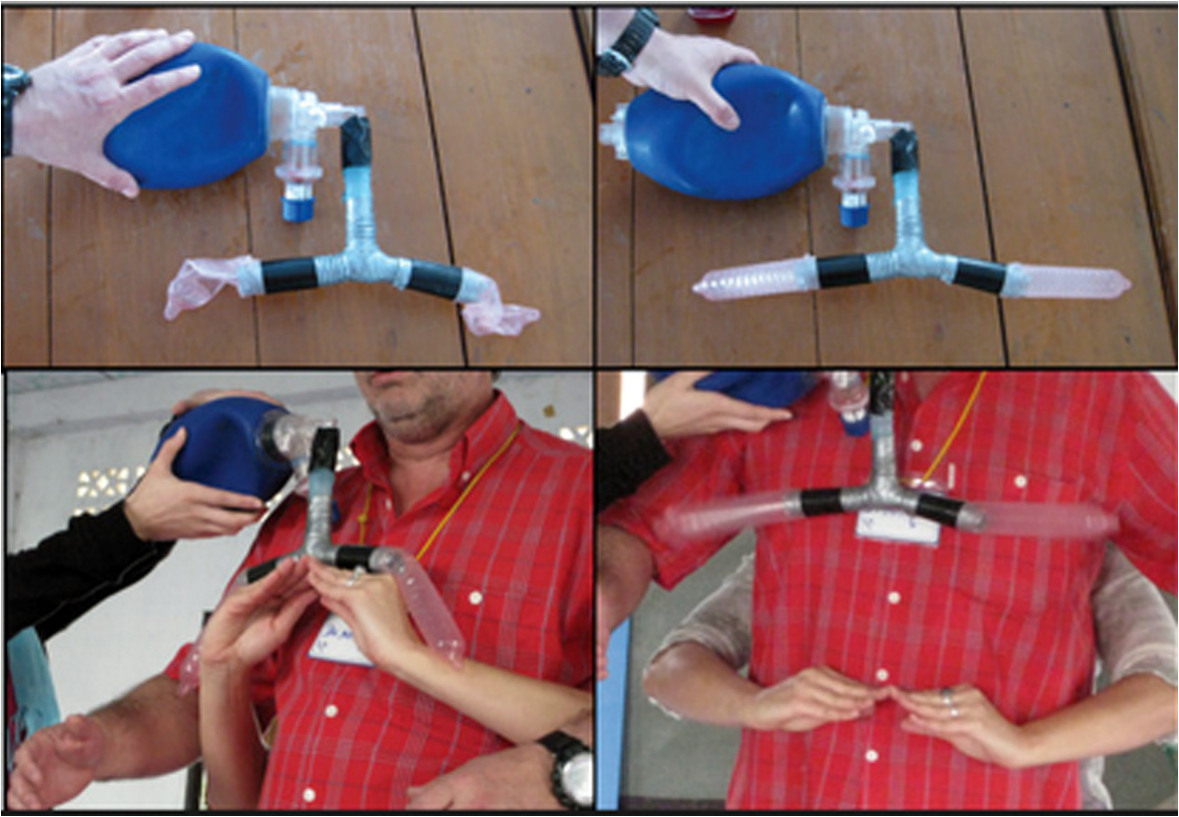
**The breathing model.** Photographs on the left represent expiration, while photographs on the right represent inspiration.

The breathing model evolved out of the necessity to teach and review basic anatomy and physiology, as the HWs have limited exposure to these topics (Figure 1). The breathing model simulates inspiration and expiration in association with diaphragm movement. The breathing model consists of a bag-valve-mask, connected to a piece of plastic respiratory tubing (representing the trachea; approximately 10 cm in length), then split into two pieces of plastic respiratory tube (representing the bronchi; approximately 7 cm in length), and then connected to a condom on each side. The model is held together with tape, and hands are used to simulate diaphragm movement.

#### Chest model

**Figure 2:**
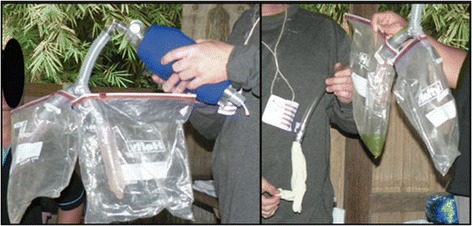
**Chest model.** The first photograph displays the chest model with normal physiology. The second photograph displays the chest model with a hemothorax and a chest tube.

The purpose of the chest model is to simulate and teach the physiology and management of a hemothorax, tension pneumothorax, and sucking chest wounds (Figure 2). The model consists of the breathing model (Figure 1) with the addition of clear plastic bags surrounding the distal plastic respiratory tubes and condoms. This model allows for the insertion of needles and chest tubes. This model does not allow for percussion, a separate model using large water bottles, which are empty, partially full, or completely full of water is used for the teaching of chest percussion (not shown).

#### Cricothyroidotomy model

**Figure 3:**
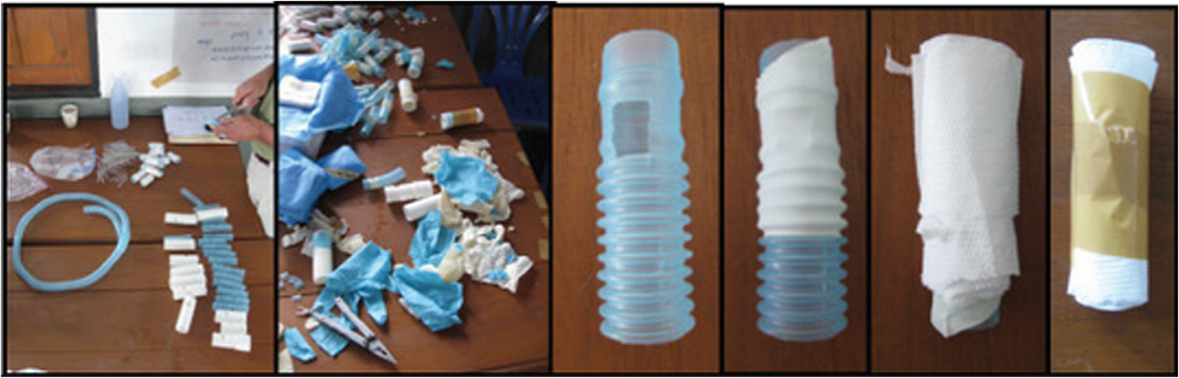
**Assembly of cricothyroidotomy model.** The first two photographs display the components, the third photograph displays the respiratory tubing with a small cutout window, which is subsequently covered with the finger of glove (fourth photograph), toilet paper (fifth photograph), and finally tape (sixth photograph).

**Figure 4:**
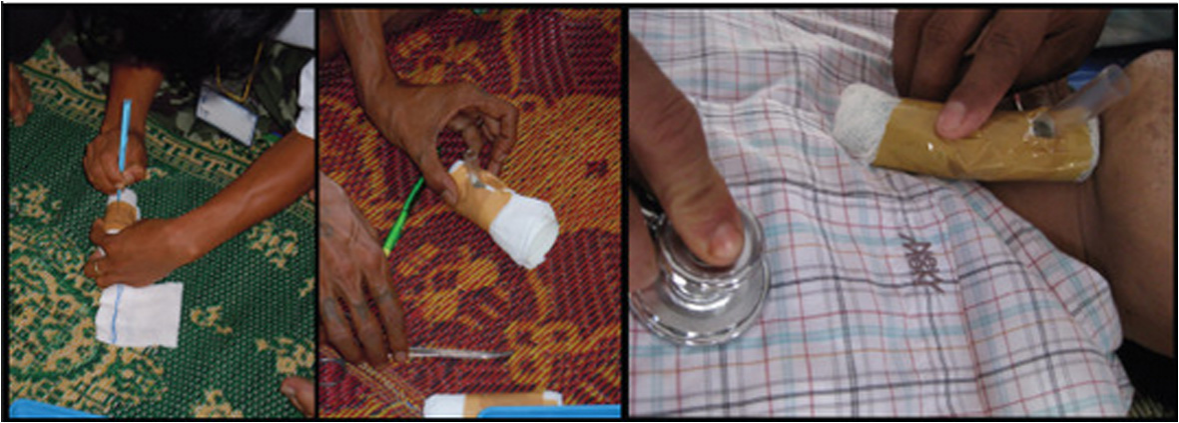
**Cricothyroidotomy model.** From left to right, the first photograph displays the initial incision, followed by insertion of the tube, and confirmation of the tube.

The cricothyroidotomy model consists of 5 cm pieces of plastic respiratory tubing with a small cutout window (approximately 1 cm by 2 cm), which is covered by a finger of a glove, toilet paper, and finally tape (Figure 3). The various layers of differing materials simulate the tissue layers of the human neck including the skin (tape), soft tissue (toilet paper), cricothyroid membrane (glove), while the larynx is represented by plastic respiratory tubing. Indications for cricothyroidotomy are discussed with the HWs. Specific to the context indications are defined as upper airway obstructions that cannot be resolved with positioning, jaw thrust, and neck extension (after determining cervical spine stability). The HWs are taught that confirmation of a correct cricothyroidotomy includes resolution of the obstruction, bilateral breath sounds, and stabilization of the patient’s vital signs (Figure 4). The HWs do not have access to oxygen or laryngoscopic capabilities; thus, this is a life saving procedure with a narrow indication and limited management alternatives.

#### Circulation model

**Figure 5:**
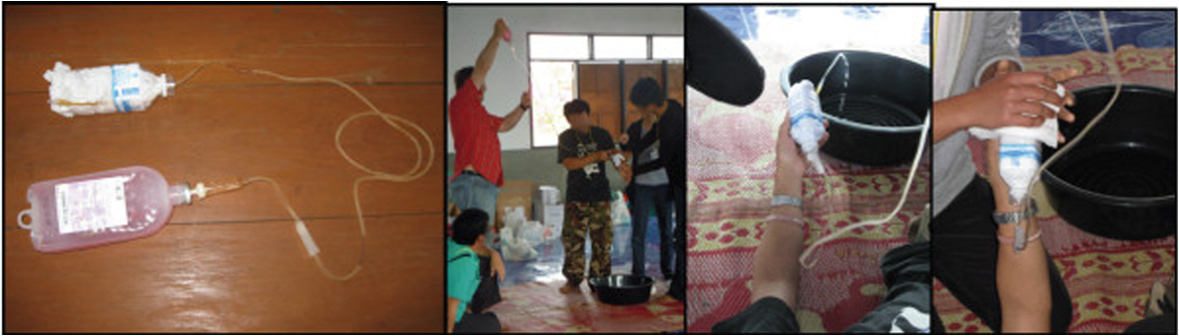
**Circulation model.** The first photograph displays the complete model, comprised of a saline bag, intravenous tubing, and a plastic water bottle stuffed with a towel. The second photograph displays pulsatile bleeding. The third and fourth photographs demonstrate the HWs managing a vascular injury with direct pressure.

The circulation model was created to simulate the vascular system and teach/practice the different procedures necessary to stop bleeding including direct pressure, elevation, packing, and application of a tourniquet (Figure 5). The circulation model is comprised of a saline bag connected with intravenous tubing to a cut plastic water bottle stuffed with a towel. The saline bag is held by the instructor, allowing him or her to produce differing amounts of pressure distally at the site of injury.

#### Wound repair model

**Figure 6:**
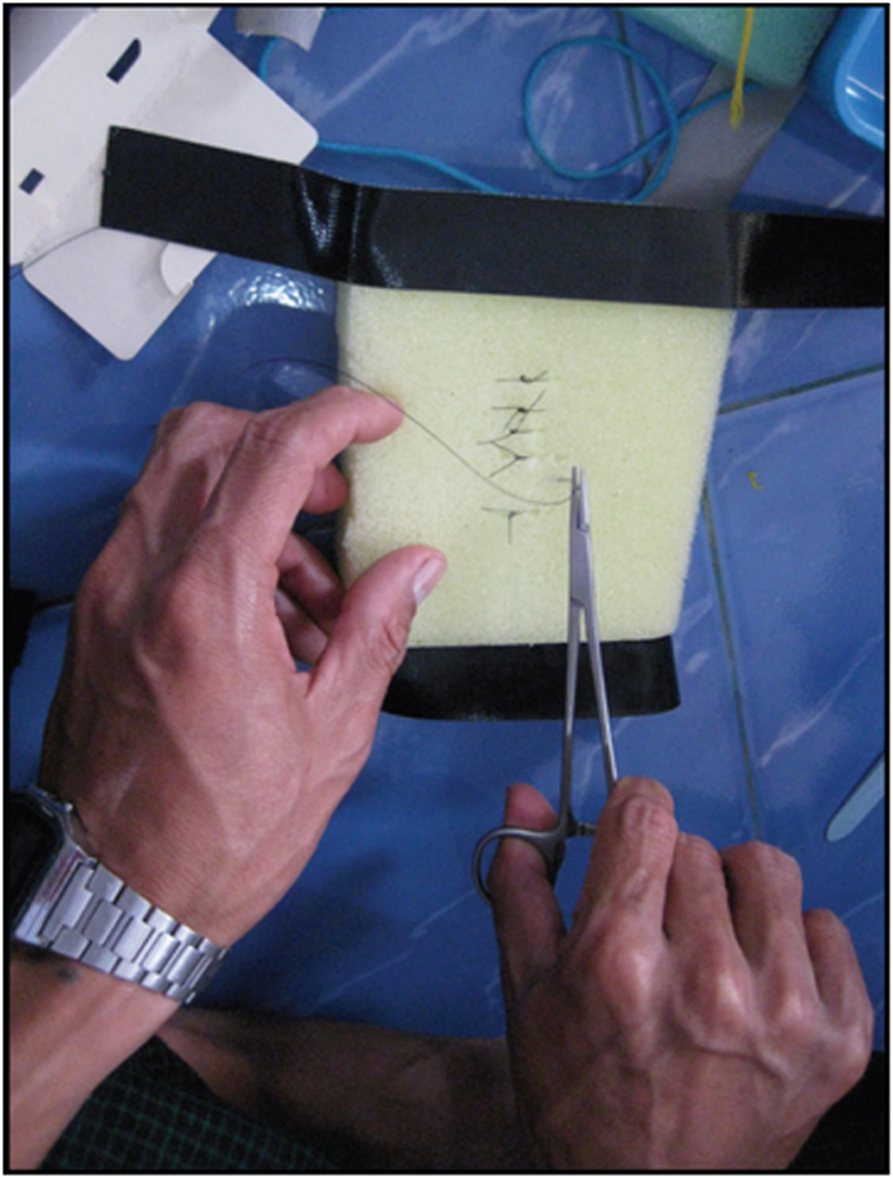
**Wound repair model.** A photograph of a HW practicing simple interrupted sutures.

The wound repair model is used to teach the techniques of wound closure, specifically the placement of simple interrupted sutures (Figure 6). Initial knot tying is taught with bicolor yarn (not shown). The wound repair model consists of a sponge with a vertical incision taped to the floor, along with sutures, a needle driver, and scissors.

#### Fracture/dislocation model

**Figure 7:**
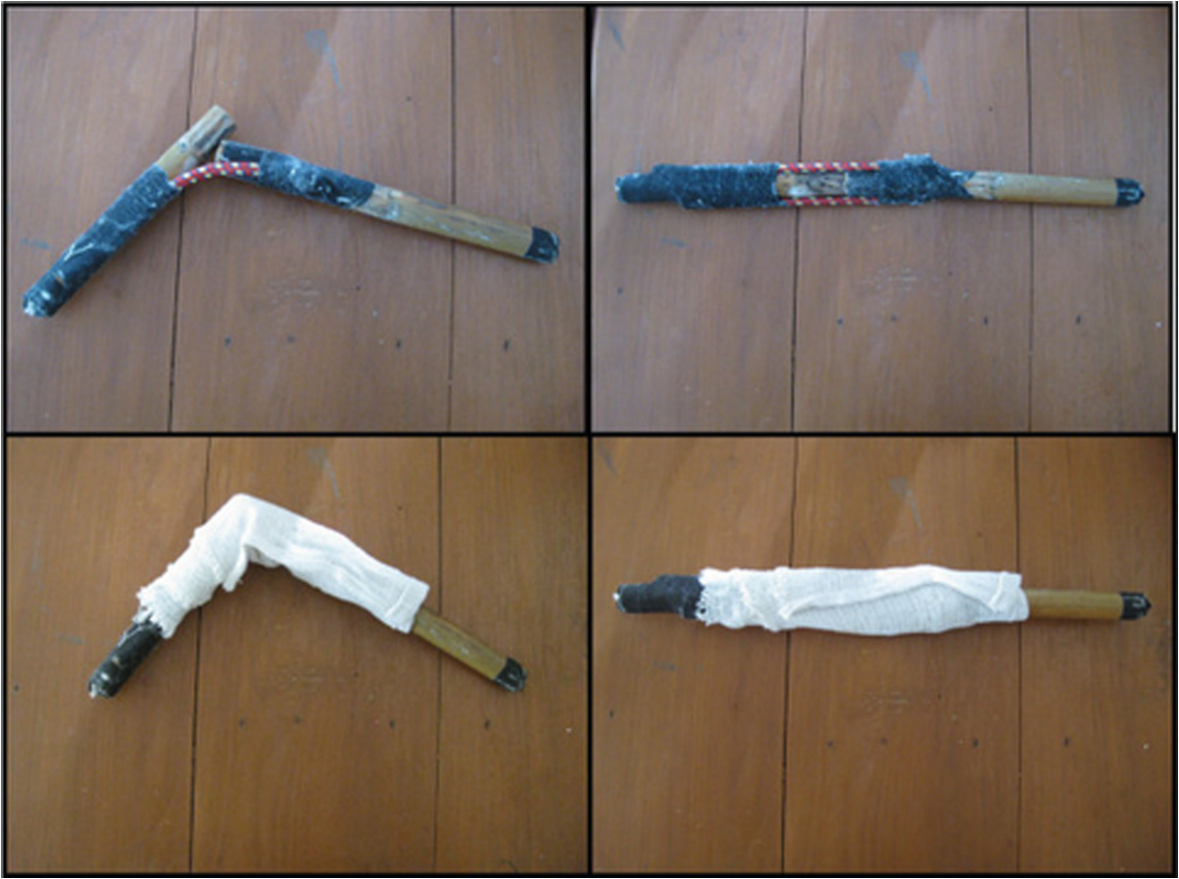
**Fracture/dislocation model.** The photographs on the left show a dislocated fracture, while the photographs on the right show a reduced fracture.

**Figure 8:**
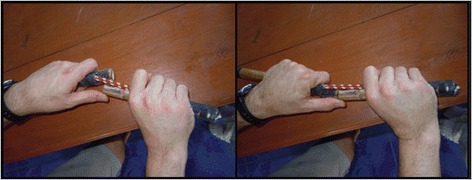
**Demonstration of one reduction method taught to the HWs with the fracture/dislocation model.**

The fracture/dislocation model demonstrates the basic physiology of a fracture/dislocation and basic techniques for reduction (Figures 7 and 8). The fracture/dislocation model consists of two pieces of sugar cane (approximately 20 cm each), two bungee cords, tape, and a small towel. The pieces of sugar cane are placed end-to-end and attached together with the two bungee cords with sufficient tension so when they are not aligned together they will form an obtuse angle. The bungee cords are attached to the sugar cane with tape. These items are wrapped in a towel to simulate soft tissue. The reduction method primarily taught is gentle traction followed by deformity exaggeration, pushing for length then reversal of the deformity.

#### Splinting

**Figure 9:**
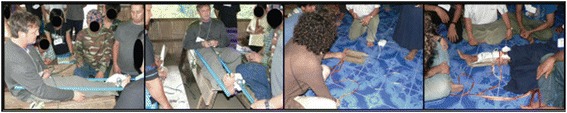
**Examples of splinting demonstrations with locally available supplies.**

Splinting is taught on human volunteers to demonstrate appropriate support and alignment (Figure 9). Locally available supplies are used, which includes rigid plastic plumbing pipes, rope, and soft fabric place mats. Trainings on plaster splints and casts are included in the TTC, as plaster is available in some of the rural clinics.

#### Fasciotomy/amputation model

**Figure 10:**
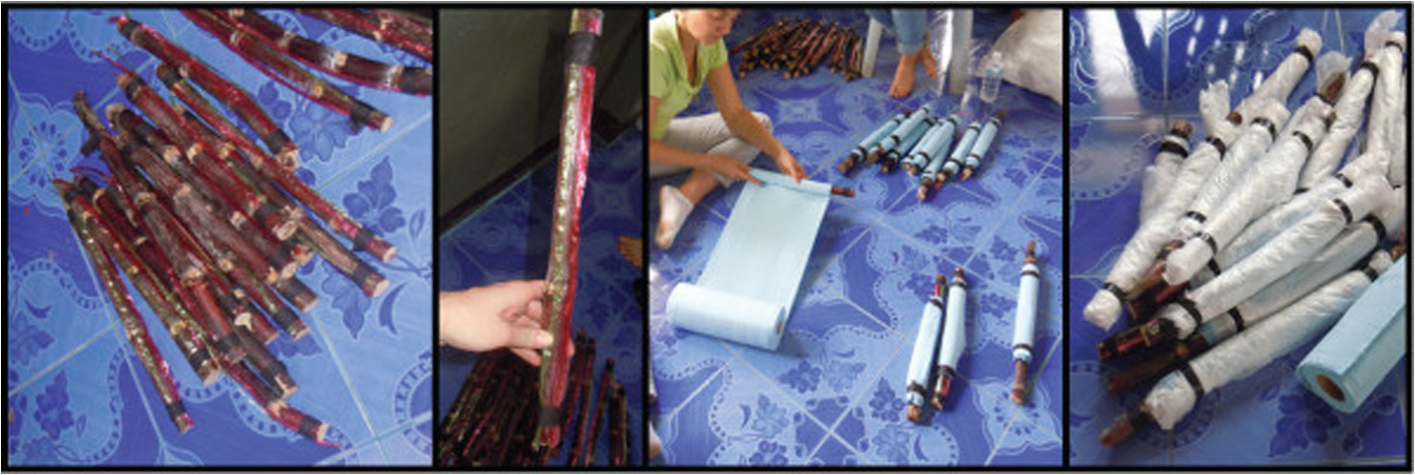
**Production of the fasciotomy/amputation model.** The first photograph displays the sugar cane pieces. The second photograph displays a sugar cane piece with two pieces of colored cellophane attached with tape. The third photograph displays the addition of paper towels to the model. The fourth photograph displays the completed model.

**Figure 11:**
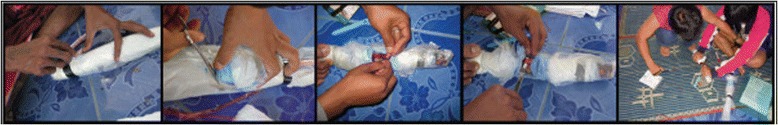
**Fasciotomy/amputation model simulation.** The first photograph displays the initial incision**.** The second photograph displays cutting and gentle dissection through the fascia and muscle layers with scissors. The third and fourth photographs display the identification, gentle dissection, and ligation of the vasculature. The fifth photograph displays cutting of the bone with a wire saw.

The fasciotomy/amputation model is used to teach basic extremity anatomy, including the relationship of the skin, subcutaneous tissue, fascia, muscle, veins, arteries, and bones (Figure 10). The model consists of approximately 1-foot-long sections of sugar cane, with two longitudinal pieces of twine (vessels that require ligation), wrapped in paper towels (muscle), and then finally two layers of plastic wrap (skin/soft tissue and fascia). The model is taught with a focus on gentle dissection including creation of a flap for skin closure after amputation, identification, and incision of the fascia, identification of arteries and veins with subsequent ligation, cutting of the bone with a hand saw, smoothing the cut end of the bone surface, wound packing, and delayed primary closure (Figure 11). Indications include blast injuries, suspected compartment syndrome, and progressive extremity infections.

#### Animal model

**Figure 12:**
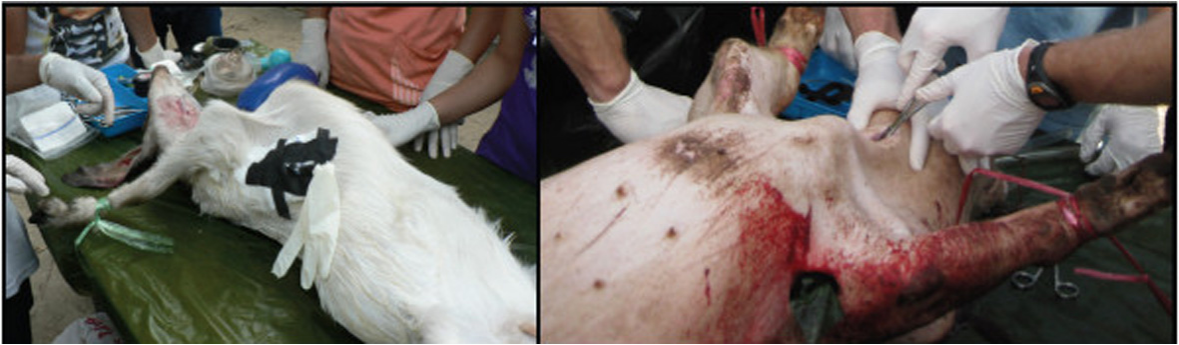
**The photographs display goat (left photograph) and pig (right photograph) animal models demonstrating cricothyroidotomy and chest tube thoracostomy placement.**

After an understanding of basic clinical skills and theory is developed by the HWs through the above listed simulation models, multiple animal simulations (pig, goat) are utilized to further solidify HWs technique and understanding (Figure 12).

#### Other teaching aids

Other teaching aids used in the TTC include a human skeleton, human simulation, and critical action cards (laminated cards that highlight the key actions for each procedure). A primary survey dance was created to reinforce the steps of primary survey (assessing for danger, the casualty’s responsiveness, airway, breathing, circulation, disability check, and exposure) in a rhythmic series of motions accompanied by chants. Stressful situations can lessen the ability to think clearly and trainers and training course directors found the dance helped HWs to recall each discrete action of the primary survey.

### Survey results

Selected cases managed by the HWs over the past year are presented in Table 2. Additionally, there were numerous reports of patients who experienced gunshot wounds or landmine injuries who were dead upon the arrival of the HWs.

**Table 2:** **Selected HW cases from 2012, including age, gender, presentation, management, and patient outcome**

**Patient**	**Case**
16-year-old male	The patient suffered a landmine injury while fishing 2 days prior to presentation. The patient had a traumatic right foot amputation. The patient subsequently was given a blood transfusion and a below the knee amputation was performed with ketamine. The patient was given antibiotics. The patient has returned to school and has been referred for a prosthesis.
18-year-old male	The patient suffered a penetrating bamboo wound to his right leg. He presented 3 days after the injury with fever, pain, erythema, swelling, and anorexia. The medic performed a fasciotomy, removed the retained bamboo, and debrided the surrounding necrotic muscle with tramadol. The patient received antibiotics and stayed at the clinic for 7 days, before being discharged.
20-year-old male	The patient was being chased by a wild boar, he climbed a tree, the branch broke and the pig bit him in his groin and legs. The patient presented to the clinic 3 h after the bite. The wounds were cleaned, packed, and he was given antibiotics.
20-year-old male	The patient suffered distal tibia and fibula fractures while playing football (soccer). The medic was on scene when the accident occurred. The medic noted the significant deformity and performed a closed reduction with tramadol. The leg was immobilized with a splint and the patient was referred for definitive management.
22-year-old soldier	The patient suffered a landmine injury. He had a complex open right ankle fracture. The wound was cleaned and packed and he was given tramadol and antibiotics. He was subsequently referred to a hospital.
24-year-old soldier	The patient suffered a gunshot wound to the right elbow. He presented to the clinic 4 h later. The wound was cleaned and the patient was given IVF and antibiotics. The medic advised an amputation, but the patient refused. The wound was debrided and packed. The patient is still unable to move his hand.
27-year-old male	The patient was gored by a buffalo and suffered multiple broken ribs and abdominal injuries. He was seen at the clinic 5 h after the injury. The patient was unconscious upon arrival to the clinic. He was given IVF and antibiotics. He was subsequently referred to a hospital.
28-year-old female	The patient suffered left open tibia and fibula fractures. The medic performed a left below the knee amputation.
28-year-old male	The patient who suffered an infected groin injury from a water buffalo 1 month prior to presentation. The patient had his penis amputated with ketamine and was given antibiotics. The patient was referred to Thailand for additional management, but he refused. On follow-up, the patient was able to urinate.
28-year-old male	The patient was cleaning a gun and it backfired, causing him an ulna fracture. The patient presented to the clinic 4 days after the injury. The medic performed a reduction with tramadol. The patient was then splinted and given antibiotics.
38-year-old male	The patient suffered a wild boar attack to his abdomen and had evisceration. The medic responded within 30 min. On arrival the patient was in severe shock and despite intravenous fluids and direct pressure the patient expired.
45-year-old male	The patient sustained left face, right chest, and left leg injuries from a wild boar attack. The patient presented 3 h after the attack. The wounds were cleaned and packed. The patient stayed in the clinic for 10 days and recovered well.

There were a number of reports of patients who experienced gunshot wounds or landmines injuries who were dead upon arrival of the medic.

A post-training evaluation was conducted in 2013, and of the 26 HWs that attended the TTC, 75% (18/24) of HWs felt confident in applying the skills they learned during the training. Almost all of the HWs felt that the training was relevant to their work (96%, 25/26), a valuable use of their time (100%, 26/26), and felt the training objectives were met (100%, 25/25). Furthermore, 96% (25/26) HWs indicated that they felt the training simulation models helped them learn and gain skills (96%, 25/26).

**Figure 13:**
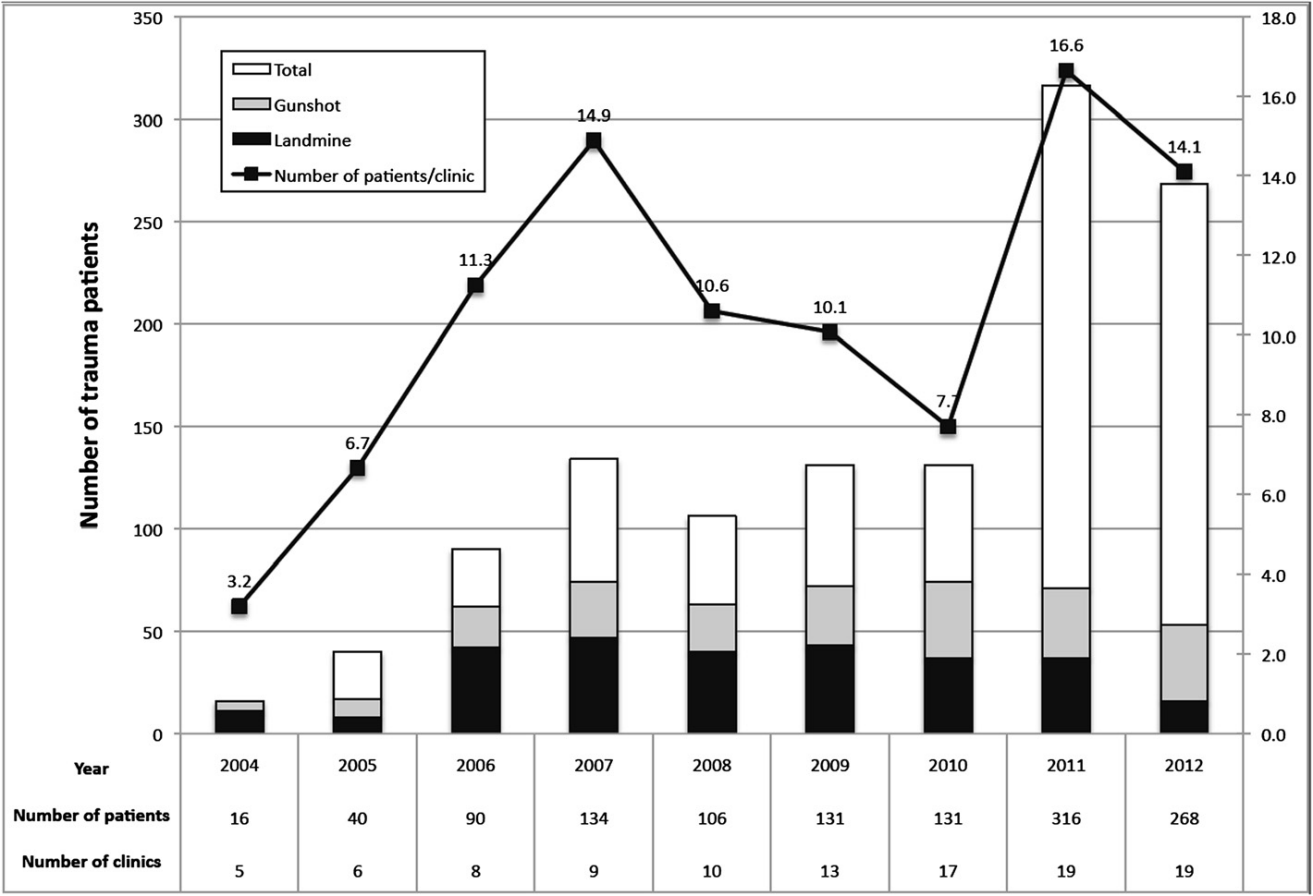
**Number of patients by year seen by the HWs involved in the trauma training course.**

Over the past 9 years, the HWs involved with the TTC have provided care to 1,232 major trauma patients, (defined as a gunshot wound, landmine, stab wound, fall from tree, a tree falling on the patient, animal bite, or problems with the patient’s airway, breathing or circulation; Figure 13). The case survival rate among major trauma patients was 94%.

## Conclusions

The TTC has continued to develop and refine trauma simulation models to facilitate an understanding of the basic knowledge necessary for the HWs to perform their skill set in a resource-limited setting. These models can be adapted and applied to the training of HWs in other settings. The simulation models described engage the HWs in clinical skills practice specific to injury management, which builds upon the HWs existing knowledge and facilitates an increased understanding of life-saving procedures. Through observation of the participant performance and obtaining feedback from HWs, trainers and training course directors have found that the simulation models increase the understanding of clinical skills of the HWs beyond what is achieved solely with a lecture-based format. Additionally, it is of paramount importance to recognize the relationship developed between the training course directors and the KDHW over the years. This longitudinal relationship has facilitated an understanding of each other’s experiences, culture, and knowledge, which serves as another conduit for adapting the training and simulation models to fit the needs of the HWs.

Limitations to the TTC have been identified. First, standard training programs for HWs are a graduated learning system starting with physiology and mechanisms of injury/disease, progressing to simulation, supervised patient care, and then finally independent practice. However in the context of the current situation in eastern Myanmar, this system is not viable. Barriers to implementation include lack of financial and human resources, ongoing armed conflict, governmental mistrust, and poor health infrastructure. Second, there are consistent requests to make the TTC longer. This is a challenge due to logistics and time constraints of the HWs and international trainers, HW coverage while attending the training, and the safety of participants as they travel across international borders. To address this, a major goal of the TTC is to transition to a training of trainers model. Currently during the trainings, senior HWs participate as clinical instructors and peer-to-peer teachers. In the future the senior HWs should teach the TTC with reduced presence of international instructors. Finally, despite 13 years of training, data regarding HWs knowledge retention and use of skills is sparse and mostly anecdotal. Improvement in data collection, including morbidity and mortality figures from the field, is currently being addressed.

The TTC has provided training to HWs in a resource-limited conflict setting over the past 13 years. We feel that every year our training course and models improve. Despite the barriers faced, we feel that this is a necessary program and has reduced morbidity and mortality in areas that the HWs serve, although the data is mostly anecdotal. With the changing political environment in Myanmar and the development of peace agreements between the government and the ethnic minority groups, these HWs can be integrated into Myanmar’s evolving health system. With their robust skill set, they can provide essential services that are unlikely to be met in the short or medium term by other providers in the rural areas they serve.
